# Phase I/II study of streptozocin monotherapy in Japanese patients with unresectable or metastatic gastroenteropancreatic neuroendocrine tumors

**DOI:** 10.1093/jjco/hyac048

**Published:** 2022-04-12

**Authors:** Izumi Komoto, Norihiro Kokudo, Taku Aoki, Chigusa Morizane, Tetsuhide Ito, Takuya Hashimoto, Wataru Kimura, Naoya Inoue, Kiyoshi Hasegawa, Shunsuke Kondo, Hideki Ueno, Hisato Igarashi, Takamasa Oono, Masatoshi Makuuchi, Takeshi Takamoto, Ichiro Hirai, Akiko Takeshita, Masayuki Imamura

**Affiliations:** 1 Department of Surgery, Kansai Electric Power Hospital, Osaka, Japan; 2 Hepato-Biliary-Pancreatic Surgery Division, Department of Surgery, University of Tokyo Hospital, Tokyo, Japan; 3 Department of Hepatobiliary and Pancreatic Oncology, National Cancer Center Hospital, Tokyo, Japan; 4 Department of Medicine and Bioregulatory Science, Graduate School of Medical Sciences, Kyushu University, Fukuoka, Japan; 5 Division of Hepato-Biliary-Pancreatic Surgery, Japanese Red Cross Medical Center, Tokyo, Japan; 6 Department of Gastroenterological, Breast, Thyroid, and General Surgery, Yamagata University Faculty of Medicine, Yamagata, Japan; 7 National Center for Global Health and Medicine, Tokyo, Japan; 8 Dokkyo Medical University Hospital, Tochigi, Japan; 9 Fukuoka Sanno Hospital, International University of Health and Welfare, Fukuoka, Japan; 10 Igarashi Medical Clinic, Yamaguchi, Japan; 11 National Hospital Organization Kyushu Cancer Center, Fukuoka, Japan; 12 Okitama Public General Hospital, Yamagata, Japan; 13 Neuroendocrine Tumor Center, Kansai Electric Power Hospital, Osaka, Japan

**Keywords:** neuroendocrine tumor, streptozocin, disease control, tumor shrinkage

## Abstract

**Background:**

This phase I/II study was conducted to evaluate the efficacy, safety and pharmacokinetics of streptozocin (STZ) in Japanese patients with unresectable or metastatic gastroenteropancreatic neuroendocrine tumors.

**Methods:**

Twenty-two patients received up to 4 cycles of intravenous STZ at either 500 mg/m^2^ once daily for 5 consecutive days every 6 weeks (daily regimen) or at 1000–1500 mg/m^2^ once weekly for 6 weeks (weekly regimen). Tumor response was evaluated using the modified RECIST criteria ver. 1.1, and adverse events were assessed by grade according to the National Cancer Institute CTCAE (ver. 4.0).

**Results:**

Fourteen (63.6%) patients completed the study protocol. No patients had complete response; partial response in 2 (9.1%), stable disease in 17 (77.3%), non-complete response/non-progressive disease in 2 (9.1%) and only 1 (4.5%) had non-evaluable disease. Excluding the latter, the response rate in the daily and weekly regimens was 6.7% (1/15) and 16.7% (1/6), respectively, with an overall response rate of 9.5% (2/21). However, the best overall response in each patient showed that the disease control rate was 100%.

Adverse events occurred in all 22 patients, including 17 grade 3 adverse events in 11 patients; however, no grade 4 or 5 adverse events were reported. Prophylactic hydration and antiemetic treatment reduced the severity and incidence of nephrotoxicity, nausea and vomiting. Plasma STZ concentrations decreased rapidly after termination of infusion, with a half-life of 32–40 min. Neither repeated administration nor dose increases affected pharmacokinetic parameters.

**Conclusions:**

STZ may be a useful option for Japanese patients with unresectable or metastatic gastroenteropancreatic neuroendocrine tumors.

## Teaser

In a phase I/II study, streptozocin in a daily or weekly regimen was effective and well-tolerated in 22 Japanese patients with unresectable or metastatic gastroenteropancreatic neuroendocrine tumors.

## Introduction

Neuroendocrine neoplasms (NENs) are rare tumors that tend to proliferate slowly. However, the number of patients with NENs is increasing. According to the second epidemiological survey conducted in Japan in 2010, the national estimates of the prevalence of gastroenteropancreatic NENs are 2.69 and 6.42 per 100 000, respectively, and annual onset incidences are 1.27 and 3.51 per 100 000, respectively (1). In a population-based study using the national cancer database of 2016, there were 6735 new cases of pancreatic and gastrointestinal NENs, with annual incidence rates of 0.70 and 2.84 per 100 000, respectively (2). Although the clinical course of well-differentiated NENs (neuroendocrine tumors: NETs) is generally indolent, NETs are frequently diagnosed at a late stage, with approximately half of patients presenting with unresectable or metastatic disease at the time of diagnosis (3), and therefore requiring chemotherapy. In Japan, the molecular-targeted drug everolimus (4) is approved for treating pancreatic and gastrointestinal NETs, the multitargeted tyrosine kinase inhibitor sunitinib (5) for treating pancreatic NETs and the somatostatin analog octreotide (6) for treating gastrointestinal NETs (GEP-NETs).

Streptozocin (STZ) is a glucosamine–nitrosourea compound derived from *Streptomyces achromogenes*, a nonmotile, aerobic, gram-positive bacterium that was approved as a cytotoxic antitumor drug for symptomatic or advanced pancreatic NETs in the USA in 1982. STZ is transported into the β-cells of pancreatic islets by the glucose transporter GLUT2, where it induces DNA strand breaks and DNA alkylation, which leads to the necrosis of pancreatic β-cells (7). In Western countries, chemotherapy with STZ in combination with doxorubicin (DOX) or fluorouracil (5-FU) is the standard of care for patients with metastatic GEP-NETs, based on several clinical trials (8–16). In Japan, a recent retrospective multicenter survey showed that STZ-based chemotherapy is effective in improving progression-free and overall survival for Japanese patients with unresectable NETs (17). Although there are reports of prior retrospective studies, no data from prospective studies are available, and there is a lack of safety evaluation and pharmacokinetic data. Therefore, we conducted a multicenter, phase I/II study to evaluate the efficacy, safety and pharmacokinetics (PK) of STZ in Japanese patients with unresectable or metastatic GEP-NETs.

## Methods

### Study design

This phase I/II, multicenter, open-label, uncontrolled study of STZ for advanced NETs (NPC-10-1) was conducted at 6 sites in Japan. Patients received up to 4 cycles of intravenous STZ, either once daily for 5 consecutive days every 6 weeks or once weekly for 6 weeks, based on treatment methods used in clinical studies outside Japan (14–16) and in Japan (17). Among the patients who completed the 4-cycle treatment, a subsequent safety assessment study was conducted in patients who wished to continue the treatment.

### Patients

Between August and December 2011, patients were enrolled in this study and assessed for eligibility. Eligible patients were Japanese adults (≥20 and <75 years of age) with a life expectancy of at least 3 months and histopathologically confirmed GEP-NET that were graded as G1 or G2 according to the World Health Organization (WHO) 2010 classification and were measurable based on the revised Response Evaluation Criteria in Solid Tumors (RECIST) guidelines (ver. 1.1) (18). Prior radiotherapy or immunotherapy was allowed if completed at least 4 and 2 months, respectively, before the start of study drug administration. Major surgery was permitted if completed at least 4 months before the start of study drug administration. Patients with previous chemotherapy, including STZ, were eligible if they had completed chemotherapy (other than STZ, DOX, antimetabolite and molecular-targeted drugs), antimetabolite therapy and chemotherapy (with DOX and/or molecular-targeted drugs) at least 4, 2 and 6 weeks, respectively, before the start of study drug administration. Given the small number of patients eligible for the study, we included 4 patients who received STZ as prior therapy to make the study viable to ensure the number of patients. Patients with only non-target lesions were also included in the study.

Other inclusion criteria were: Eastern Cooperative Oncology Group (ECOG) performance status of 0–2; neutrophils ≥1500/mm^3^, hemoglobin ≥9.0 g/dL and platelets ≥100 000/mm^3^; total bilirubin ≤1.5 × the institutional upper limit of normal (ULN), AST and ALT ≤2.5 × ULN (AST or ALT ≤5 × ULN in patients with liver involvement); serum creatinine ≤1.5 × ULN; BUN ≤30 mg/dL and electrocardiogram readings within ranges not requiring treatment. Patients were required to be hospitalized for up to 8 days from the start of study drug administration.

Patients were excluded if they had small or large cell neuroendocrine carcinoma, mixed adenoneuroendocrine carcinoma, and hyperplastic and preneoplastic lesions according to the WHO 2010 classification, or if there was evidence of an active infection. Other exclusion criteria included severe or uncontrolled complications of heart disease (including congestive heart failure, poorly controlled angina or arrhythmia), myocardial infarction within 3 months of study registration, poorly controlled hypertension, poorly controlled diabetes, liver disease (including cirrhosis, chronic active hepatitis or chronic persistent hepatitis), renal disease (including acute or chronic renal failure), lung disease (including interstitial pneumonia, pulmonary fibrosis or severe emphysema), active hemorrhagic diatheses (including coagulation disorders associated with abnormal platelet and/or coagulation factors, requiring supplementation of platelets and/or coagulation factors, or treatments such as corticosteroids), symptomatic primary or metastatic brain tumor, clinically significant fluid retention (including pleural effusion, drainage, peritoneal fluid, pericardial effusion), participation in other clinical trials within 3 months before study registration, long-term use of systemic corticosteroids or other immunosuppressants, duplicate cancers, current pregnancy or breast feeding, female patients who wished to become pregnant during and 3 months after the trial period, male patients who did not agree to use adequate contraception, such as the barrier method, and baseline abnormalities or medical conditions that could jeopardize the patient’s safety or interfere with the study.

### Drug administration

Patients naïve to STZ received up to 4 cycles of intravenous STZ at a dose of either 500 mg/m^2^ for 0.5–2 h once daily for 5 consecutive days (days 1–5) every 6 weeks (daily regimen) or at a dose of 1000 mg/m^2^ for 0.5–2 h once weekly for 6 weeks (weekly regimen) until disease progression, unacceptable toxicity or withdrawal of consent without dosage increase or reduction. Patients with prior STZ treatment received up to 4 cycles of the weekly regimen, in which the initial dose of 1000 mg/m^2^ was increased to 1250 mg/m^2^ at week 13 and further to 1500 mg/m^2^ at week 19 or decreased to a minimum level of 750 mg/m^2^ if dose reduction was necessary. Patients could receive octreotide in combination with STZ when it was used previously but could not receive combination therapy (including chemotherapy, interferon preparations, hormone therapy, immunotherapy, radiation therapy, surgery or other investigational drugs) because it could alter the effects of STZ.

To prevent anticipatory nausea and vomiting and reduce risk of nephrotoxicity associated with STZ, patients could receive adequate hydration and antiemetic treatment before and during each STZ regimen.

### Evaluation of efficacy and safety

Tumors were evaluated by imaging (CT or MRI) before and 6 weeks after the start of each STZ regimen. The tumor shrinkage rate (best observed response) from baseline was determined according to the RECIST criteria (ver. 1.1). Response rate was defined as the ratio of complete response (CR) and partial response (PR) of the best observed response, and disease control rate was defined as the percentage of CR, PR and stable disease (SD). For the assessment of safety, adverse events (AEs) were recorded from the start of the initial STZ regimen to the end of the trial. During the hospital stay and at each visit, subjective symptoms and objective findings were obtained by medical examination (including interviews, visual inspection and auscultation), electrocardiograms and clinical examinations. Severity was determined based on the Common Terminology Criteria for Adverse Events (CTCAE, ver. 4.0).

### Pharmacokinetics

Blood samples were collected from all subjects for PK measurements. Before and during each STZ regimen, patients received the following treatment: (i) electrolyte infusion (500 mL) for 120 min, (ii) electrolyte infusion (100 mL) + antiemetic for 30 min, (iii) electrolyte infusion (100 mL) + STZ for 30 min and (iv) electrolyte infusion (250 mL) for 60 min. Patients in the daily regimen had blood collected before each STZ infusion on days 2–4 of the first cycle for trough value measurement, before STZ infusion, immediately before the end of STZ infusion, 10, 20, 30, 60, 90 min, and 3 and 6 h after STZ infusion on days 1 and 5 for full PK measurements. Patients in the weekly regimen had blood samples collected for full PK measurements before STZ infusion, immediately before the end of STZ infusion and 10, 20, 30, 60, 90 min, and 3, 6, and 24 h after the end of STZ infusion on Day 1 of the first cycle and the third or fourth cycle after dose increase.

### Statistical analysis

Analyses were performed on the following sets: (i) safety analysis population; (ii) full analysis set (FAS: largest analysis population); (iii) per protocol set (population meeting the protocol) and (iv) pharmacokinetic analysis population. To determine the efficacy of the treatment regimens, the tumor shrinkage (best overall effect), response and disease control rates were analyzed based on the RECIST criteria (ver. 1.1). For safety, the frequency and incidence of AEs were analyzed by grade based on CTCAE (ver. 4.0). Plasma concentration–time data were used to calculate PK parameters.

### Rationale for setting the number of patients

Based on the 36% response rate after STZ monotherapy reported by Moertel *et al.* (8), the expected response rate for the present study was set at 40%. The threshold response rate of this study was set at 5%, based on the threshold response rate of 5% in overseas phase II studies of everolimus and sunitinib malate in pancreatic NETs (4,5). With a 5% threshold response rate on the antitumor effect of STZ and an expected response rate of 40%, a sample size of 10 patients naïve to STZ (in the daily regimen) was required to ensure that the lower limit of the 95% confidence interval (CI) of the response rate would exceed the threshold response rate at a significance level of 5%, with a detection power of 80%. In addition, a survey on the actual use of STZ in Japan conducted prior to this study (17) indicated that STZ had been administered to 10 patients when this study was being planned. Therefore, the sample size of patients with prior STZ treatment (in the weekly regimen) was set at 6 after considering feasibility.

### Ethics and consent acquisition

This clinical trial was conducted according to the criteria stipulated in the ‘Ethical Principles Based on the Declaration of Helsinki’, ‘Pharmaceutical Affairs Law Article 14, Paragraph 3 and Article 80-2 (1954 Law No. 145)’ and in compliance with the ministerial ordinance (GCP) on the implementation standard (March 27, 1997 Ministry of Health Ordinance No. 28). All documents and materials pertinent to this clinical trial are appropriately stored at each responsible department. In addition, the protocol of this study was reviewed and approved by the institutional review committee of each medical institute. Prior to implementing the study, the principal (shared) investigator adequately used explanatory documents and other appropriate materials to explain the trial process to patients and obtained a voluntarily signed informed consent form for participation in the trial.

## Results

### Patient characteristics

**Figure 1:**
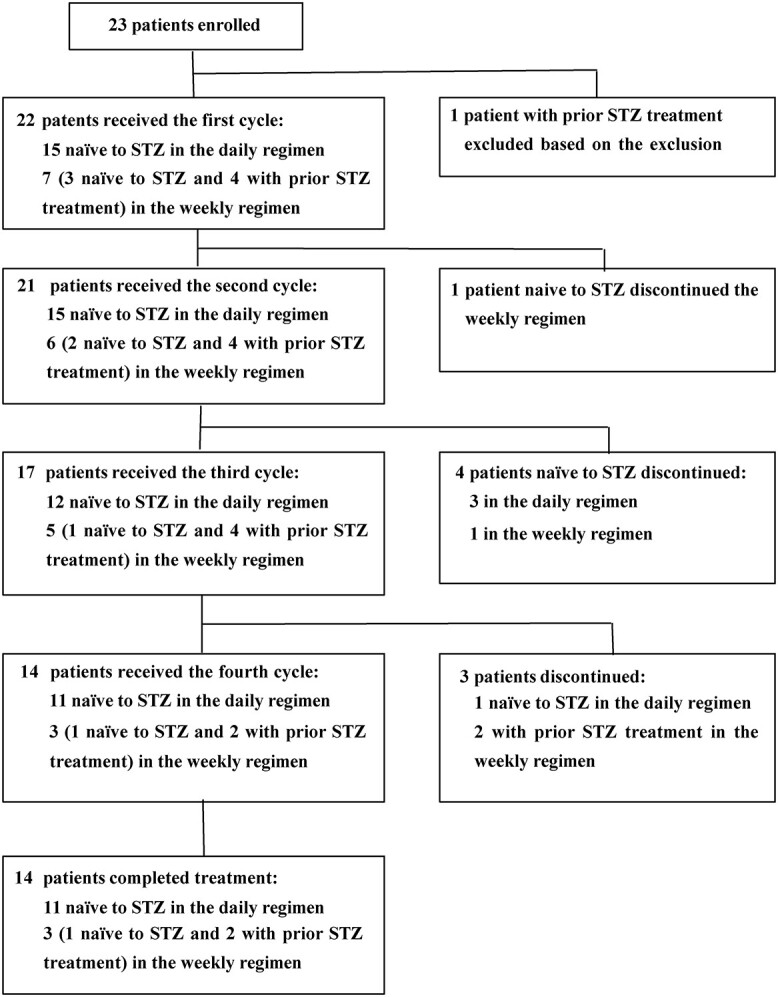
A trial profile showing the number of patients enrolled, the number of patients who received the daily and weekly regimens, the number of patients who discontinued and the number of patients who remained in the study. *STZ,* streptozocin.

Of 23 enrolled patients, one patient was excluded based on exclusion criteria during the screening period, and the remaining 22 were evaluable for efficacy and safety. As shown in Fig. 1, 15 patients naïve to STZ were allocated to the daily regimen, and 3 naïve to STZ and 4 with prior STZ treatment were allocated to the weekly regimen. Overall, 14 patients, including 12 naïve to STZ and 2 with prior STZ treatment, received up to 4 cycles of STZ treatment and completed the study. Of 22 patients, 22 completed cycle 1, 21 completed cycle 2, 17 completed cycle 3 and 14 completed cycle 4. The number of patients who discontinued treatment was 4 in the daily regimen; 3 at the end of cycle 2 (due to worsening of general condition, worsening of diabetes and PD) and 1 at the end of cycle 3 (due to PD). In the weekly regimen, 4 patients discontinued the study; 1 at the end of cycle 1 (due to AE of neutropenia), 1 at the end of cycle 2 (due to PD) and 2 at the end of the cycle 3 (due to PD).

The cumulative dose of STZ in all patients was 18095.6 ± 11096.5 mg (mean ± standard deviation): 14270.5 ± 3931.5 mg in the daily regimen and 26292.3 ± 16731.2 mg in the weekly regimen. The dose intensity in all patients was 119.57 ± 63.24 mg/day: 90.53 ± 12.35 mg/day in the daily regimen and 181.80 ± 83.76 mg/day in the weekly regimen.

Patient demographics and baseline characteristics of the 22 patients are listed in Table 1. There were 10 men (45.5%) and 12 women (54.5%) with a mean age (±standard deviation) of 56.8 ± 7.1 years. In tumor functionality, 5 (22.7%) patients had functional pancreatic NETs, including 5 (22.7%) with gastrinoma and 1 (4.5%) of them with complicated somatostatinoma, and 12 (54.5%) had non-functional pancreatic NETs. In primary tumor lesions, 15 (68.2%) patients had pancreatic NETs, 4 (18.2%) had gastrointestinal NETs, 2 (9.1%) had GEP-NETs and 1 (4.5%) had an unknown primary lesion. According to the 2010 WHO classification for primary lesions, 5 (22.7%) patients had G1 NETs, 15 (68.2%) had G2 NETs and 2 (9.1%) had G1 and G2 NETs. In patients with gastrointestinal NETs, lesions were found in the foregut, midgut and hindgut in 5 (22.7%), 1 (4.5%) and 1 (4.5%) patient, respectively. There was a history of excision of the primary tumor in 12 (54.5%) patients. Metastatic lesions were found in all patients; the 2 most common metastatic sites were the liver and lymph nodes, occurring in 21 (95.5%) and 11 (50.0%) patients, respectively. There was a history of metastatic lesion resection in 10 (45.5%) patients. Gastrin overproduction was observed in 4 (18.2%) patients. There was a history of chemotherapy with or without somatostatin in 18 (81.8%) patients. None of the patients had prior radiotherapy and/or immunotherapy. The ECOG PS during the screening period was 0 and 1 in 20 (90.9%) and 2 (9.1%) patients, respectively.

**Table 1:** Patient demographics and disease characteristics (full analysis set, *n* = 22)

Parameter		Total	Daily	Weekly
**Number of patients**		22	15	7
**Age in years**	Median (range)	59 (43–68)	61 (43–68)	54 (48–60)
**Sex**	Male (%)	10 (45.5)	7 (46.7)	3 (42.9)
	Female (%)	12 (54/5)	8 (53.3)	4 (57.1)
**Primary tumor lesion**	Pancreatic NET (%)	15 (68.2)	9 (60.0)	6 (85.7)
	Gastrointestinal tract NET (%)	4 (18.2)	4 (26.7)	0 (0.0)
	Pancreatic/gastrointestinal tract NET (%)	2 (9.1)	2 (13.3)	0 (0.0)
	Unknown NET (%)	1 (4.5)	0 (0.0)	1 (14.3)
**Tumor functionality**	Functional (%)	5 (22.7)^a^	3 (20.0)^a^	2 (28.6)
	Nonfunctional (%)	12 (54.5)^b^	8 (53.3)^b^	4 (57.1)
**Tumor grade**	Grade 1 (%)	5 (22.7)	3 (20.0)	2 (28.6)
	Grade 2 (%)	15 (68.2)	10 (66.7)	5 (71.4)
	Grades 1 and 2 (%)	2 (9.1)	2 (13.3)	0 (0.0)
**Primary lesion excised (%)**		12 (54.5)	8 (53.3)	4 (57.1)
**Metastatic site**	Liver (%)	21 (95.5)	14 (93.3)	7 (100.0)
	Lung (%)	3 (13.6)	3 (20.0)	0 (0.0)
	Lymph nodes (%)	11 (50.0)	8 (53.3)	3 (42.9)
	Abdominal cavity (%)	2 (9.1)	2 (13.3)	0 (0.0)
	Others (%)	3 (13.6)	1 (6.7)	2 (28.6)
**Metastatic lesion excised (%)**		10 (45.5)	7 (46.7)	3 (42.9)
**Previous treatment**	Surgery (%)	8 (36.4)	6 (40.0)	2 (28.6)
	Radiation (%)	0 (0.0)	0 (0.0)	0 (0.0)
	Systemic chemotherapy and/or somatostatin analogue (%)	18 (81.8)	13 (86.7)	5 (71.4)
**Performance status**	0 (%)	20 (90.9)	13 (86.7)	7 (100.0)
	1 (%)	2 (9.1)	2 (13.3)	0 (0.0)
	2–4 (%)	0 (0.0)	0 (0.0)	0 (0.0)

^a^Duplication of functional pancreatic and gastrointestinal NETs (subject code: C-03; daily regimen).

^b^Duplication of nonfunctional pancreatic and gastrointestinal NETs (subject code: C-02; daily regimen).

### Efficacy

**Figure 2:**
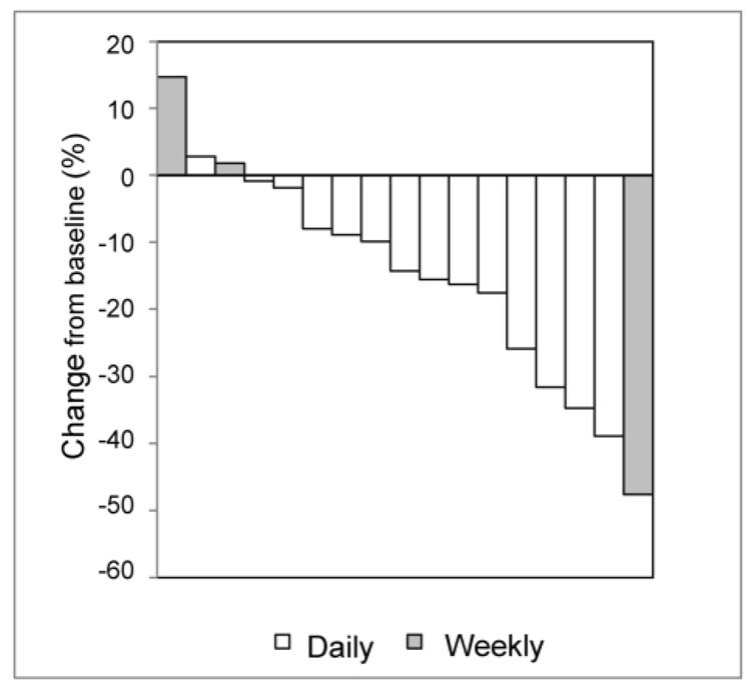
A waterfall plot of changes in tumor size from baseline in the STZ-naïve patients (14 in the daily regimen and 3 in the weekly regimen). Shaded bars represent patients receiving the weekly regimen.

Clinical efficacy was analyzed in all 22 patients who received at least one cycle of STZ infusion (FAS). The best observed responses in these 22 patients are shown in Table 2. No patients achieved CR. PR was observed in 2 (9.1%) patients, SD in 17 (77.3%) and non-CR/non-progressive disease (PD) in 2 (9.1%). None of the patients had PD, and 1 (4.5%) patient had non-evaluable (NE) disease. However, the evaluable 21 (100%) patients had achieved effective disease control. When responses to each of the 2 regimens were compared, 1 of 15 (6.7%) patients and 1 of 7 (14.3%) patients in the daily and weekly regimens, respectively, achieved PR. SD was observed in 13 of 15 (86.7%) and 4 of 7 (57.1%) patients, and non-CR/non-PD was observed in 1 of 15 (6.7%) and 1 of 7 (14.3%) patients in the daily and weekly regimens, respectively. One STZ-naïve patient each in the daily and weekly regimens achieved PR. Two patients in the daily regimen, whose comprehensive evaluations at the end of the trial indicated PR, had primary non-functional and functional pancreatic NETs, both of which had been previously treated with everolimus and the fluoropyrimidine anticancer drug TS-1®. Figure 2 shows a waterfall plot of maximum tumor shrinkage from baseline in the 17 STZ-naïve patients (14 in the daily regimen and 3 in the weekly regimen). Although the degree of shrinkage varied, target tumor shrinkage was observed in 14 of 17 patients.

**Table 2:** Best overall response according to RECIST criteria

	Total	Daily	Weekly	
		Naïve to STZ	Naïve to STZ	Prior STZ treatment
Number of patients	22	15	3	4
CR^a^	0 (0.0)	0 (0.0)	0 (0.0)	0 (0.0)
PR	2 (9.1)	1 (6.7)	1 (33.3)	0 (0.0)
SD	17 (77.3)	13 (86.7)	1 (33.3)	3 (75.0)
Non-CR/Non-PD	2 (9.1)	1 (6.7)	0 (0.0)	1 (25.0)
PD	0 (0.0)	0 (0.0)	0 (0.0)	0 (0.0)
NE	1 (4.5)	0 (0.0)	1 (33.3)	0 (0.0)

*Naïve to STZ*: no history of streptozocin treatment, *Prior STZ treatment*: history of prior streptozocin treatment, *CR* complete response, *PR* partial response, *SD* stable disease, *PD* progressive disease, *NE* not evaluable.

^a^Number of patients (%).

### Safety

All 22 patients (15 and 7 in the daily and weekly regimens, respectively) who received at least one STZ treatment were included in the safety analysis set. In total, 396 AEs were reported in 22 (100%) patients; 209 occurred in the daily regimen and 187 in the weekly regimen. No grade 4 or 5 AEs were observed, and 17 grade 3 AEs were reported, including liver enzyme elevation in 6 (27.3%) patients, lymphopenia in 2 (9.1%), leukopenia in 1 (4.5%), nausea in 1 (4.5%), hyperglycemia in 1 (4.5%), diabetes in 1 (4.5%), gum infection in 1 (4.5%), duodenal ulcer in 1 (4.5%), acute cholangitis in 1 (4.5%), joint exudate in 1 (4.5%) and hypertension in 1 (4.5%). Serious AEs were acute cholangitis and duodenal ulcer, of which duodenal ulcer had a relationship with the study drug. The duodenal ulcer occurred on day 184 (>2 weeks after the end of 4 cycles of the weekly regimen) and disappeared after hospitalization and blood transfusion. AEs leading to the discontinuation of treatment included grade 2 diabetes and grade 2 neutropenia in 1 patient each (4.5%); however, a causal relationship with STZ was denied in both cases. No grade 4 or 5 AEs were observed and 17 grade 3 AEs were reported. The reported grade 3 AEs include increased γ-GTP in 4 (18.2%) patients, decreased lymphocyte count in 2 (9.1%) patients, and gingival infections, diabetes mellitus, hyperglycaemia, hypertension, duodenal ulcer, nausea, acute cholangitis, joint effusion, increased ALT, increased AST and decreased white blood cell count each in 1 (4.5%) patient (Table 3). There was no increase in the number of AEs in patients undergoing more prolonged treatment (i.e. those who received 3 and 4 cycles). The incidence of AEs in patients with increasing doses (*n* = 3) was 51, 35 and 37 events at 1000, 1250 and 1500 mg, respectively, suggesting no tendency toward increased severity of AEs with increasing dose in the weekly regimen.

**Table 3:** Grade 3 adverse events in STZ treatment

	Total *n* = 22	Daily regimen *n* = 15	Weekly regimen *n* = 7
Gingival infections	1 (4.5)	1 (6.7)	0
Diabetes mellitus	1 (4.5)	1 (6.7)	0
Hyperglycaemia	1 (4.5)	1 (6.7)	0
Hypertension	1 (4.5)	1 (6.7)	0
Duodenal ulcer	1 (4.5)	0	1 (14.3)
Nausea	1 (4.5)	1 (6.7)	0
Cholangitis acute	1 (4.5)	1 (6.7)	0
Joint effusion	1 (4.5)	1 (6.7)	0
ALT increased	1 (4.5)	0	1 (14.3)
AST increased	1 (4.5)	0	1 (14.3)
γ-GTP increased	4 (18.2)	2 (13.3)	2 (28.6)
Lymphocyte count decreased	2 (9.1)	2 (13.3)	0
White blood cell count decreased	1 (4.5)	0	1 (14.3)

Number of patients with AE (%).

### Pharmacokinetics

**Figure 3:**
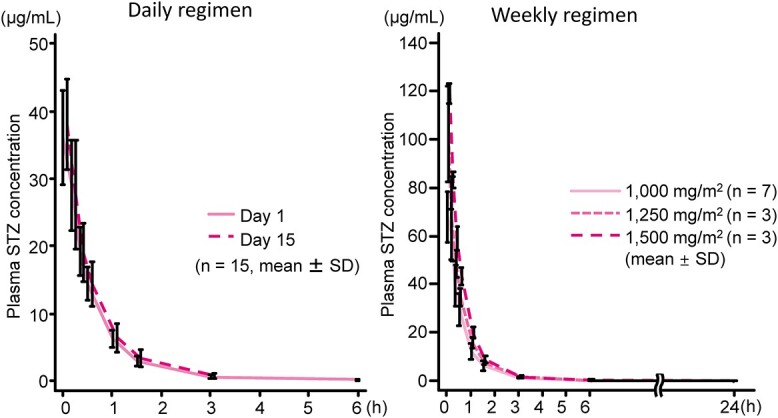
Plasma concentration of STZ in patients after intravenous infusion.

Plasma STZ concentrations were measured in all 22 patients. In both regimens, plasma STZ concentrations were highest immediately before the end of infusion, disappeared by 1.5 h after the end of infusion and mostly remained below the quantification limit (20 ng/mL) after 3 h. Table 4 and Figure 3 show PK parameters in patients after intravenous infusion of STZ. In the daily regimen, systemic exposure (maximum plasma drug concentration [*C*_max_] and area under the plasma drug concentration–time curve [AUC]) to STZ was similar on days 1 and 5, whereas in the weekly regimen, systemic exposure to STZ increased with increasing doses of STZ over the dose range of 1000–1500 mg/m^2^. The terminal phase half-life of STZ was 32–40 min in both regimens, which is comparable to the 35–40 min reported in studies outside Japan (19).

**Table 4:** Pharmacokinetic parameters of STZ in patients after intravenous infusion

Regimen	Measurement day	Dose (mg/m^2^)	*n*	*C*_max_ (μg/mL)	AUC_0–∞_ (μg·h/mL)	*t*_1/2_ (h)
Daily	Day 1	500	15	36.6 ± 6.8	31.2 ± 5.0	0.615 ± 0.056
	Day 5	500	15	39.4 ± 8.2	33.3 ± 6.9	0.665 ± 0.086
Weekly	Week 1	1000	7	68.4 ± 9.5	63.4 ± 10.2	0.637 ± 0.046
	Week 13	1250	3	102.3 ± 20.0	81.5 ± 11.8	0.604 ± 0.033
	Week 19	1500	3	119.0 ± 4.1	97.3 ± 5.4	0.546 ± 0.055

*STZ*, streptozocin; *C*_max_, maximum plasma drug concentration; *AUC_0–∞_*, area under the plasma drug concentration–time curve from 0 to infinity, *t*_1/2_ terminal phase half-life.

## Discussion

We evaluated the efficacy and safety of STZ in Japanese patients with unresectable or distant metastatic GEP-NETs in up to 4 cycles (24 weeks) of a daily or weekly regimen. The overall response rate in the 21 evaluable patients, based on the RECIST criteria (ver. 1.1), was 9.5% (95% CI: 1.2–30.4). The response rate in the STZ-naïve patients was 6.7% (1/15) in the daily regimen and 33% (1/3) in the weekly regimen, and the response rate in patients with prior STZ treatment was 0% (0/4).

In establishing the target number of patients in the present study, the threshold response rate was set at 5%, with reference to clinical trials outside Japan of molecular-targeted drugs in pancreatic NETs. In the present study, the lower limit of the 95% CI was lower than the threshold response rate. The efficacy judgment period of this study was up to the end of 4 cycles, but evaluation at the end of 4 cycles revealed that 2 patients achieved PR. A subsequent safety assessment study was conducted in 9 patients who completed the 4-cycle treatment and wished to continue the treatment. During this subsequent safety study for ≥2 years, PR was observed in 2 additional patients on STZ therapy. Therefore, STZ therapy may provide efficacy in Japanese patients with GEP-NETs similar to that in patients outside Japan.

Kouvaraki et al. (14) reported that NETs respond moderately to chemotherapy with 5-FU, DOX and STZ and that there are cases with delayed onset of effect; they recommended that patients who achieved SD after 2 cycles of treatment should continue to receive at least 4 cycles. Turner et al. (16) reported that 1 patient had SD after the end of the treatment with 5-FU, cisplatin and STZ and achieved PR at the subsequent follow-up. Approximately one-fifth of the response examples tend to develop with a delayed effect. In tumors where there is a delay in the onset of efficacy, it has been suggested that sensitivity to chemotherapeutic agents (chemosensitivity) may be underestimated (16). Weatherstone et al. (15) reported that NETs, unlike other cancer types, respond to cytotoxic agents slowly, based on similar delayed responses in at least 2 studies (14,16), suggesting the possibility of underestimation if there is no follow-up investigation. Based on these reports, as GEP-NETs have a moderate response to chemotherapy and the onset of its effect is delayed, patients with SD at the end of treatment are recommended to receive additional doses. Similar results in patients received ≥4 cycles of treatment in our study that also supports this conclusion. In double-blind comparative studies of everolimus, sunitinib malate, octreotide acetate and lanreotide acetate in patients with pancreatic NET or GEP-NET, the best observed response rates according to the RECIST criteria were 4.8% (4), 9.3% (5), 2.4% (6) and 2% (20), respectively, which are similar to the best observed response rate of 9.5% in the present study, suggesting that similar efficacy can be expected.

Nephrotoxicity, nausea and vomiting are the main AEs of STZ, which are areas of concern according to the literature from outside Japan (8,14–16,21). In the present study, all patients received prophylactic hydration and antiemetic agents (5HT_3_ receptor antagonists, NK_1_ receptor antagonists and dexamethasone in various combinations (22)) during each STZ regimen. Despite a few cases of mild-to-moderate proteinuria and several cases of increased blood creatinine, no grade 3 nephrotoxicity was observed, which suggested that our preventative therapy against nephrotoxicity was effective. Nausea occurred in 12 (54.5%) patients in the daily and weekly regimens, whereas vomiting occurred in 5 (22.7%) patients only in the daily regimen. Therefore, these indicate that prophylactic hydration and antiemetic treatment reduced the severity and incidence of nephrotoxicity and nausea/vomiting, respectively.

In this study, we evaluated the PK profile of STZ in patients with GEP-NET. In both regimens, plasma STZ concentrations were highest immediately before the end of infusion, decreased by 1.5 h after the end of infusion and mostly remained below the quantification limit after 3 h. The terminal phase half-life of STZ was 32–40 min after the end of STZ infusion, which is similar to the 35–40 min reported in overseas studies (19). STZ PK parameters were not affected by repeated administration or dose increases, and STZ accumulation was not observed.

In recent years, everolimus (4) and octreotide (6) were approved for pancreatic and gastrointestinal NETs, respectively. It has been proposed that the chemotherapy drugs should be selected according to disease progression and tumor volume (23,24). For treatment of pancreatic or gastrointestinal NETs, somatostatin may be used when the tumor size is small and disease progression is slow, molecular-targeted agents should be selected when the tumor size is medium, and cytotoxic agents including STZ are preferred when the tumor size is bulky and disease progression is fast (23,24). In our study, the efficacy and safety of daily and weekly STZ regimens were not significantly different. Because of the small number of cases in the weekly regimen, it is difficult to conclude which is better, the daily regimen or the weekly regimen.

The limitation of this study is that the present clinical trial was an open-label, uncontrolled study with a small number of cases. Therefore, large-scale placebo-controlled randomized clinical studies should be conducted on STZ alone and in combination with DOX or 5-FU because the use of STZ in combination with DOX or 5-FU has not been established in Japan.

In this phase I/II study of STZ, tumor shrinkage was observed in Japanese patients with unresectable or metastatic GEP-NETs, confirming that the efficacy of STZ in Japanese patients is similar to that reported in Caucasian patients. In addition, the safety was similar to that reported outside Japan and was within an acceptable range. Importantly, AEs were generally predictable and measures to prevent and manage them can be established on an individual basis. Therefore, STZ is expected to broaden the range of treatment for unresectable or metastatic GEP-NETs with controllable adverse effects.
